# Implications of Aquaglyceroporin 7 in Energy Metabolism

**DOI:** 10.3390/ijms19010154

**Published:** 2018-01-04

**Authors:** Francesco Maria Iena, Janne Lebeck

**Affiliations:** Department of Biomedicine, Aarhus University, Wilhelm Meyers Allé 3, 8000 Aarhus, Denmark; francescomaria.iena@biomed.au.dk

**Keywords:** aquaglyceroporins, obesity, insulin resistance

## Abstract

The aquaglyceroporin AQP7 is a pore-forming transmembrane protein that facilitates the transport of glycerol across cell membranes. Glycerol is utilized both in carbohydrate and lipid metabolism. It is primarily stored in white adipose tissue as part of the triglyceride molecules. During states with increased lipolysis, such as fasting and diabetes, glycerol is released from adipose tissue and metabolized in other tissues. AQP7 is expressed in adipose tissue where it facilitates the efflux of glycerol, and AQP7 deficiency has been linked to increased glycerol kinase activity and triglyceride accumulation in adipose tissue, leading to obesity and secondary development of insulin resistance. However, AQP7 is also expressed in a wide range of other tissues, including kidney, muscle, pancreatic β-cells and liver, where AQP7 also holds the potential to influence whole body energy metabolism. The aim of the review is to summarize the current knowledge on AQP7 in adipose tissue, as well as AQP7 expressed in other tissues where AQP7 might play a significant role in modulating whole body energy metabolism.

## 1. Introduction

Thus far, 13 members of mammalian aquaporins (AQPs) have been identified (AQP0–12), and most of them have been well characterized. In general terminology, the AQP family can be divided into three major subgroups based on their permeability characteristics and amino acid sequence homology. AQP0, AQP1, AQP2, AQP4 and AQP5 belong to the group of classical *aquaporins*, also called *orthodox aquaporins* that show water permeation only [1]. The second group, called *aquaglyceroproteins,* is permeable to small uncharged molecules, such as urea or glycerol in addition to water. AQP3, AQP7, AQP9 and AQP10 belong to this subgroup [1]. AQP6, AQP8, AQP11 and AQP12 belong to the third subgroup called *unorthodox aquaporins* whose function is still being elucidated [1]. This review will focus on the aquaglyceroporin AQP7 and its role as a facilitator of glycerol transport. AQP7, previously also named AQPap [2,3,4] and even AQP9 [5], was first identified in rat testis [6]. The rat *aqp7* gene encodes a 269 amino acid (aa) protein, with a predicted molecular mass of 28.9 kDa [6]. The deduced amino acid sequence of human AQP7 (hAQP7) is 342 aa long [7,8], while in mice (mAQP7), the protein is 303 aa in length [8]. The hAQP7 sequence is 67% identical to mAQP7, and both share 68% and 79% sequence homology with rat AQP7 (rAQP7), respectively. AQP7 shares the overall structure of other AQPs, being arranged into six transmembrane domains with N- and C-termini facing the cytosol. The two classical asparagine–proline–alanine (NPA) motifs at the core of the AQPs are, however, not fully conserved in AQP7. In hAQP7 and mAQP7, the first NPA motif is substituted by a NAA (asparagine–alanine–alanine) motif, while the second NPA motif is NPS (asparagine–proline–serine) [5,8]. In rats, the first NPA motif is preserved, but the the second NPA motif is NPS [6]. Experiments in *Xenopus oocytes* expressing AQP7 showed increased water permeability [3,5,6], along with glycerol and urea uptake [6]. Similar results have been obtained in 3T3-L1 adipocytes for glycerol and water [9,10]. *Auphen*, a water-soluble gold(III) compound that exerts inhibitory effects on AQP3, also inhibits AQP7 function [9]. AQP7 is expressed in a wide range of tissues and this review will focus on the tissues where current knowledge suggests a role for AQP7 in whole body energy metabolism. 

## 2. Adipose Tissue

Adipose tissue is the main tissue involved in storage of body fat, and has in more recent years emerged as an organ with important endocrine functions [11]. According to energy requirements, adipocytes regulate their triglyceride content in response to hormonal and neural stimulation. During energy depletion, triglycerides (TGs) are hydrolyzed into free fatty acids (FFA) and glycerol, which are both utilized by other tissues to meet energy needs. In this context, AQP7, as a glycerol channel in adipose tissue, plays a significant role in determining glycerol availability in both adipose tissue and in the circulation [12].

### 2.1. Localization of AQP7 in Adipose Tissue

**Figure 1:**
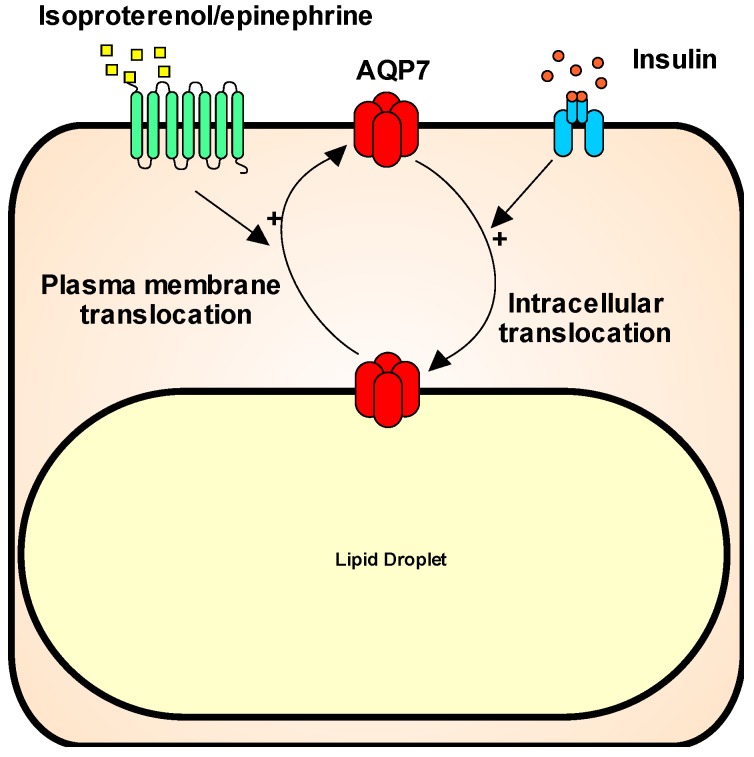
Schematic illustration of the proposed trafficking of AQP7 in adipocytes. Isoproterenol and epinephrine acting likely through beta-adrenergic receptors promotes trafficking of AQP7 from the lipid droplet to the plasma membrane. The opposite translocation occurs in response to insulin stimulation.

AQP7 is abundantly expressed in human and rodent adipose tissue [4,5,8,13,14]. In rodents, AQP7 is found in both white and brown adipose tissue [8,15]. The cellular localization of AQP7 in adipose tissue has been the subject of some debate; however, in white adipose tissue (WAT), the evidence points towards AQP7 being expressed in both adipocytes and in capillary endothelial cells in both mice [10,16] and humans [17]. In vitro studies in the mouse embryonic fibroblast/adipose tissue-like cell line (3T3-L1) [2,18] and human adipocytes [17] have suggested a dynamic localization of AQP7 in adipocytes, where AQP7 supposedly traffics between an intracellular localization and the plasma membrane in response to feeding and fasting. Specifically, translocation from intracellular compartments to the plasma membrane has been observed upon epinephrine/isoproterenol stimulation [2,17,18], while insulin has been proposed to stimulate internalization [17,18] (Figure 1). Recently, in vitro studies in human primary adipocytes has suggested that, in response to insulin stimulation, AQP7 localization to the plasma membrane is prevented by binding to the lipid droplet (LD)-associated protein perilipin 1 (PLIN1). In response to isoprenaline, the binding of AQP7 to PLIN1 is inhibited by phosphorylation of the cytosolic N-terminus of human AQP7 at S10/T11 by protein kinase A, thus allowing the translocation of the protein to the plasma membrane [7]. Interestingly, this phosphorylation site resides at the initial part of the AQP7 N-terminus, which is only found in human AQP7 [8]. In contrast to these results, Miyauchi and coworkers reported the opposite effects of insulin and norepinephrine on AQP7 localization in mouse adipocytes, with insulin promoting a cortical distribution of AQP7 and norepinephrine stimulating translocation of AQP7 to LDs [16]. Moreover, leptin has also been found to promote translocation of AQP7 from LDs to the plasma membrane domain [19]. In all, these interesting in vitro findings call for further studies to confirm whether such trafficking occurs in vivo. Furthermore, clarification of which stimuli promote trafficking to and from the LDs/plasma membrane is warranted. 

### 2.2. Regulation of AQP7 in Adipose Tissue

A negative insulin response element has been identified in the promotor region of the mouse [3] and human [4] *aqp7* gene. In WAT from male mice, the AQP7 expression is inversely regulated by insulin acting through the phosphatidylinositol 3-kinase (PI3K) pathway [3]. This results in increased expression during fasting and decreased expression in the fed state [3], thus paralleling the changes in lipolytic rate during these states. Increased abundance of AQP7 has been found in response to streptozotocin- [3,15] and diet- [20] induced diabetes mellitus, as well as in insulin resistant db+/db+ [21] and ob/ob mice [19]. In contrast to the line of results mentioned above, in vitro stimulation of human omental adipocytes with insulin was reported to increase the abundance of AQP7 [18]; thus, further studies are needed to clarify whether species-specific regulation of AQP7 exists. 

Peroxisome proliferator-activate receptors (PPARs) are a group of nuclear receptors that act as transcription factors regulating gene expression. The peroxisome proliferator activated receptor γ (PPARγ) regulates adipocyte differentiation and the expression of several adipose tissue-related genes upon binding to a peroxisome proliferator response element (PPRE) in the promotor region of the target gene [22]. PPARγ agonists stimulate storage of TG in adipose tissue [23] in part by stimulating glycerol kinase (GlyK) activity [24], which increases the phosphorylation of glycerol into glycerol-3-phosphate, which can be utilized for TG synthesis. A PPRE has been identified in the promotor of both mouse and human AQP7 [4,13] and PPARγ agonist treatment results in increased WAT AQP7 mRNA abundance in male rodents [13,25]. The parallel increase in AQP7 and GlyK activity could thus promote the uptake of glycerol for TG synthesis in adipocytes. Similar to PPARγ, activation of both PPARβ/δ [26] and PPARα [27] have been reported to increase the abundance of AQP7 mRNA in adipocyte cell culture models.

The plasma concentrations of the adipokine leptin are positively correlated with body fat mass [28]. It exerts an anti-obesity effect in healthy individuals [29], in part by binding to receptors highly expressed in the hypothalamus, where it triggers molecular signaling that regulates food intake and controls energy homeostasis [30]. Leptin receptors are also found in peripheral tissues, including WAT [31]. In response to chronic leptin administration, a reduced AQP7 abundance was found in both wild-type (WT) and leptin-deficient ob/ob male mice [19]. This was paralleled by a marked reduction in WAT mass and lower plasma levels of FFA and glycerol, the latter suggesting that the reduced WAT AQP7 abundance was paralleled by a decreased lipolytic rate after 4 weeks of treatment. Similar results were obtained by in vitro studies in human [18] and mouse [19] adipocytes, suggesting a direct effect of leptin on WAT AQP7 expression.

Ghrelin, a peptide hormone synthesized by cells lining the stomach, plays a major role in the short-term regulation of appetite, and in contrast to leptin, it enhances appetite [32]. In vitro stimulation of adipocytes from human omental WAT with ghrelin resulted in an increased accumulation of triglyceride (TG), along with a reduced AQP7 expression and an increased expression of lipogenic enzymes. This observation is in line with ghrelin inducing adiposity and reduced fat utilization in rodents [33].

In addition to the regulatory mechanisms mentioned in the above, WAT AQP7 expression is also influenced by sex. Men have a lower AQP7 mRNA abundance in both subcutaneous and visceral WAT than women [34], and the response to exercise training is sex-dependent, causing an increased AQP7 expression in subcutaneous WAT in women, whereas in men the opposite result was observed [35]. Moreover, an association between obesity and a single nucleotide polymorphism (SNP) in the promotor of AQP7 causing a decrease transcription of the *aqp7* gene was found only among women [36]. How this sexual dimorphism in the regulation of WAT AQP7 expression occurs remains to be elucidated. In support for estradiol promoting WAT AQP7 expression high fat diet (HFD) fed ovariectomized mice showed a decreased abundance of AQP7 in visceral (but not subcutaneous) WAT which was reversed by estradiol supplementation [37]. However, further studies are needed to identify whether the effect was due to an indirect or direct effect of estradiol on WAT. Moreover, in adipocytes from early pregnant rats an increased WAT AQP7 expression in parallel with increased glycerol uptake and increased GlyK activity was reported when compared to control rats [38].

Finally, decreased expression of AQP7 mRNA has been reported in response to isoproterenol, TNFα, and dexamethasone administration in cell culture models, whereas no effect on AQP7 mRNA abundance was observed upon exposure to angiotensin 2, growth hormone, triiodothyronine, epinephrine, glucagon, and ACTH [2,39]. Isoproterenol is a selective β-agonist that stimulates lipolysis, and it therefore seems counterintuitive that it would decrease the abundance of AQP7. Thus, further studies are needed to clarify this matter, as well as how this agrees with the proposed role in AQP7 trafficking. 

### 2.3. Role of AQP7 in Adipose Tissue

As outlined above, AQP7 function in adipose tissue is mainly linked to its ability to facilitate glycerol transport across cell membranes. This agrees with most findings suggesting that regulation of AQP7 abundance and AQP7 trafficking to the plasma membrane domain parallels the synthesis of glycerol from TG lipolysis. In adipose tissue, the activity of GlyK is negligible, and therefore glycerol needs to exit the adipose tissue in order to be metabolized by other tissues. Whether AQP7 is a significant regulator of adipose tissue glycerol efflux has been investigated in AQP7 knockout (KO) mice [15,40,41,42,43]. Importantly, characterization of the different AQP7 KO mouse lines has not all resulted in the same conclusions. Furthermore, all studies have been performed in whole-body KO models and, as outlined below, the lack of AQP7 in other tissues, such as pancreatic β-cells, muscle and kidney has the potential to influence whole body energy metabolism. In the mouse line generated by Maeda and coworkers [42], fed and fasted male AQP7 KO mice presented lower p-glycerol levels and higher glycerol concentrations in WAT than WT littermates. Furthermore, the AQP7 KO mice responded to fasting with more marked hypoglycemia, plausibly as a result of an impaired de novo synthesis of glucose from glycerol. Further characterization of this AQP7 KO mouse line showed that after 12 weeks of age, AQP7 KO mice developed adult-onset obesity with adipocyte hypertrophy and whole-body insulin resistance [41]. A marked increase in WAT GlyK activity was found in AQP7 KO, suggesting that higher intracellular glycerol concentrations leads to increased GlyK activity and in turn increases the synthesis of TG. Another AQP7 deficiency mouse model also demonstrated glycerol accumulation in WAT, leading to adipocyte hypertrophy at 16 weeks of age [40]. Here, body weight gain was similar to WT mice; however, AQP7 KO mice had markedly increased body fat mass, but a shorter body length compared to WT controls. Adipocyte hypertrophy or increased body fat mass were not reported in the phenotyping of two other AQP7 KO mouse lines [15,43].

### 2.4. AQP7 in Human Adipose Tissue

The proposed linkage between AQP7 deficiency and obesity with secondary development of insulin resistance has also been investigated in human studies. In support for such an association, AQP7 was identified as one of the dysregulated adipose tissue genes in obese humans [44], and increased expression of AQP7 mRNA was found in peripheral blood of a small cohort of obese men and women when compared to matched controls [45]. In addition, when evaluating the association between obesity and genome-wide DNA methylation in subcutaneous adipose tissue, the *aqp7* gene was identified as one of methylated genes associated with obesity [46]. In addition, the human *aqp7* gene is localized in a chromosomal region with reported linkage to the metabolic syndrome [47] and type-2-diabetes (T2D) [48,49]. As mentioned above, a SNP (A953G) in the promoter of AQP7 that causes decreased transcriptional activity is associated with obesity and secondary development of T2D in women, thus to some extent mimicking the observations made in AQP7 KO mice [41]. A male subject homozygous for a loss of function mutation in AQP7 (G264V) did not present an apparent metabolic phenotype [4]; and similarly, no association between the G264V mutation and obesity or T2D was found in a cohort of 178 Caucasians. However, in this cohort, only one subject was homozygous for the mutation and this individual was obese with type 2 diabetes and low p-glycerol levels [50]. Moreover, homozygosity for the G264V mutation in AQP7 was identified in 3 unrelated children (1 girl and 2 boys) with psychomotor retardation, normoglycerolemia, hyperglyceroluria, and a mild defect in platelet secretion. No apparent metabolic phenotype was observed in either the 3 affected children or their normal homozygous siblings or heterozygous parents [51]. In all, the potential role of AQP7 deficiency in increased susceptibility to development of obesity seems most evident among women, which pinpoints that future studies should be performed in a gender-specific manner.

In other human studies, the relative AQP7 abundance has been evaluated in adipose tissue from lean, obese individuals, as well as individuals with T2D with conflicting results, and, except for one study [52], all were performed in mixed-gender cohorts. Several studies found reduced AQP7 mRNA expression in subcutaneous WAT from obese individuals [18,50,53]. However, unchanged AQP7 mRNA abundance in subcutaneous WAT has also been reported [54]. Intriguingly, gastric bypass surgery in morbidly obese men and women resulted in a decreased expression of AQP7 mRNA in subcutaneous WAT 6 and 12 months after surgery [55]. In contrast to the observations made in subcutaneous WAT, increased AQP7 gene expression was found in visceral WAT, when comparing obese with overweight individuals [54]. Most of the studies evaluating AQP7 mRNA expression in subcutaneous or visceral WAT in association with T2D did not find any significant changes [50,52,54,56]; however, increased AQP7 abundance in visceral WAT from type 2 diabetics has been shown [18,54]. A significant increase in visceral WAT AQP7 expression was also found in a small cohort of insulin-resistant women with polycystic ovary syndrome (PCOS) characterized by endocrinological dysfunction, hyperandrogenism, absence of ovulation and polycystic ovaries [57]. In summary, taking into account that these studies were not designed to identify if a changed AQP7 abundance would be a cause or a consequence of obesity or T2D, and that most of them were conducted in mixed gender cohorts, the results agree with an association between reduced AQP7 abundance and obesity, whereas a direct association between dysregulated AQP7 abundance and T2D seems less evident.

### 2.5. Other Aquaporins Expressed in Adipose Tissue

As evaluated by northern blotting, no AQP3 or AQP9 were found in mouse WAT or brown adipose tissue [2,42]. In human subcutaneous WAT, no or only low levels of the other aquaglyceroporins; AQP3, AQP9 and AQP10 were detected using Q-PCR, and the mRNA levels of AQP3, AQP9 and AQP10 remained unaffected in individuals hetero- or homozygous for the G264V mutation, suggesting no compensatory regulation of these aquaglyceroporins in response to AQP7 loss of function [54]. Moreover, as evaluated by immunohistochemistry, no AQP3 [35,58] or AQP9 protein [35] were detected in human WAT. However, in recent studies, the presence of AQP3, AQP9 and AQP10 has been reported in cultured adipocytes and human WAT [17,18,59].

The expression of AQP7 in adipose tissue endothelial cells overlaps with AQP1 [15,60], and in starved AQP7 KO mice, an increased expression of WAT AQP1 protein was found [60], thus suggesting a role for AQP7 in adipose tissue water homeostasis.

In addition, expression of the unorthodox AQP11 has been identified in human and mouse adipocyte cell lines, where an intracellular localization near lipid droplets was reported. Overexpression of human AQP11 in 3T3-L1 adipocytes was reported to increase the plasma membrane permeability of water and glycerol [59]; how this correlates with the reported intracellular localization of AQP11 remains to be determined. Moreover, previous investigations of rat and human AQP11 expressed in *Xenopus oocytes* found no permeability to water, and no glycerol or urea was detected [61]. Further studies are needed to clarify the potential functional overlap of other aquaporins with AQP7. 

## 3. Kidney

AQP7 is abundantly expressed in the kidney, where it is localized in the brush border plasma membrane of the proximal tubule (segment 3) and to a lesser extent in the proximal convoluted tubules [14,15,35,62]. Here, it is poorly involved in water reabsorption; instead, it plays a major role in glycerol reabsorption [63], with AQP7 KO mice presenting marked glyceroluria [15,63]. Despite the marked urinary loss of glycerol, plasma glycerol levels remained unaffected in these AQP7 KO mouse lines [63]. The marked urinary loss of glycerol and thus energy would oppose development of obesity and if the reabsorbed glycerol is used for renal gluconeogenesis [64] this could compromise renal glucose production or shift the balance towards usage of other gluconeogenic precursors. In support for a similar function of renal AQP7 in humans, the three unrelated children previously mentioned, being homozygous for the G264V mutation, showed normal plasma glycerol levels along with a marked glyceroluria [51].

## 4. Muscle

AQP7 expression is found in both skeletal [15,65] and cardiac muscle [15,66]. In skeletal muscle, AQP7 has been localized to the capillary endothelial cells [15] as wel as localization to the myocyte sarcolemma also have been reported [67]. In obese leptin deficient ob/ob mice, the skeletal muscle AQP7 expression is increased compared to WT mice [68]. With glycerol being an important precursor for intramuscular TG synthesis [69,70], it could be speculated that the increased expression of AQP7 in obese mice would supply glycerol for an increased lipid storage in skeletal muscle. Further studies are needed to clarify the role of AQP7 in skeletal muscle.

In cardiomyocytes, the most common sources of energy are fatty acids and glucose [71]. However a significant role for glycerol in cardiomyocyte energy metabolism has also been proposed [72,73]. Like in skeletal muscle, AQP7 expression is localized to the capillary endothelial cells in mouse heart [15]; however, AQP7 mRNA expression has also been found in H9c2 myocytes derived from embryonic rat heart [73]. AQP7 deficient mice had a significant reduction in cardiac glycerol consumption and ATP content, thus indicating a role for AQP7 in controlling cardiac glycerol metabolism and ATP production [73]. Moreover, the AQP7 KO mice were more susceptible to development of left ventricle hypertrophy and had an increased mortality rate in different models of cardiac stress [73]. In all, these results support a significant role for AQP7 in controlling cardiac glycerol metabolism. Moreover, studies in rats suggest that the cardiac expression of AQP7 is increased in response to ischemia [74] exercise [75,76] and high-protein diet [76].

## 5. Endocrine Pancreas

AQP7 is expressed in the β-cells in the islets of Langerhans of both rats and mice, where it is localized to intracellular domains [43,77]. Despite its apparent cytosolic localization, studies in AQP7 KO mice suggest a role for AQP7 in modulating the intraislet glycerol concentration [43]. AQP7-deficient mice presented an increased intraislet glycerol concentration and increased GlyK activity, thus mirroring the results obtained by Hibuse and coworkers in WAT from AQP7 KO mice [41]. Moreover, the AQP7 KO mice had reduced β-cells mass and increased proinsulin biosynthesis with marked fasting hyperinsulinemia. Intriguingly, the latter was paralleled by normal plasma glucose levels, with no sign of insulin resistance [43]. Normally, GlyK activity in pancreatic β-cells is negligible; however, when genetically introduced, glycerol has been identified as a stimulator of proinsulin biosynthesis and insulin secretion [78,79], thus suggesting that the induction of GlyK activity is central to observations made in AQP7 KO mice by Matsumara and coworkers. In contrast to these results, no hyperinsulinemia was found when investigating the role of AQP7 in pancreatic β-cells in the AQP7 KO mouse line where adipocyte hypertrophy [40] and marked glyceroluria [63] had previously been demonstrated. Furthermore, in this study a reduced insulin secretion was observed in islets isolated from AQP7 KO mice in response to rising d-glucose concentrations, extracellular hypotonicity and isosmotic addition of glycerol as compared to WT islets [80]. AQP7 deficiency was found to be associated with impairment of β-cell swelling and changes in membrane potential in response to glycerol administration, thus proposing an alternative mechanism for AQP7 influencing insulin secretion [80]. Recently, the pancreatic expression of AQP7 was reported to be increased in HFD fed obese rats, and when evaluating the effect of sleeve gastrectomy as a surgical intervention for the amelioration of obesity, a further increase in pancreatic AQP7 expression was observed. Moreover, using rat RIN-m5F insulinoma β-cells, it was found that both acetylated ghrelin and glucagon-like peptide 1 (GLP1) decreased the abundance of AQP7, whereas ghrelin had no effect on insulin secretion and GLP1 increased insulin secretion [81]. In all, further studies are needed to clarify how changes in pancreatic AQP7 expression influence insulin secretion. 

## 6. Liver

AQP9 is considered the main facilitator of glycerol uptake in hepatocytes [1,82,83]. AQP7 expression has also been documented in both human [18,67,84,85,86] and mouse [67,84,87] liver. However, others have reported no AQP7 expression in murine [3,14,15,82] and rat [6] liver, and studies of human AQP7 have indicated no [4] or very low levels of AQP7 mRNA in liver [85]. In human liver, AQP7 has been localized to the cytoplasmic domain of hepatocytes [84,88] and the apical plasma membrane domain of cholangiocytes and endothelial cells [84]. In mice immunohistochemical staining was observed just in the hepatocyte cytoplasmic domain [84]. In all, the cellular and subcellular localization of AQP7 in liver does not point towards a functional overlap with AQP9, and further studies are needed to clarify the potential role of AQP7 in liver.

## 7. Conclusions

Accumulating evidence supports the notion that AQP7 is a glycerol channel with significant influence on glycerol availability in the tissues it is expressed in. In adipose tissue, AQP7 deficiency is linked to increased TG accumulation and development of obesity; however, when considering targeting of AQP7 in anti-obesity therapy, the wide range of tissues where AQP7 is expressed should be taken into account. As summarized here, AQP7 plays a pivotal role in renal glycerol reabsorption. The increased expression of AQP7 in skeletal muscle from obese mice could support increased lipid accumulation, and thus promote skeletal muscle insulin resistance and in cardiac muscle, AQP7 seems to play a significant role in energy metabolism. Finally, despite conflicting results, AQP7 expressed in pancreatic β-cells seems to influence insulin secretion, which in turn would modulate whole-body energy metabolism. In conclusion, AQP7 is a significant modulator of glycerol metabolism in a wide range of tissues with implications for whole-body energy balance as well as the pathophysiology of obesity and development of insulin resistance.
